# Comparison of chicken 7SK and U6 RNA polymerase III promoters for short hairpin RNA expression

**DOI:** 10.1186/1472-6750-7-79

**Published:** 2007-11-19

**Authors:** Stephanie C Bannister, Terry G Wise, David M Cahill, Timothy J Doran

**Affiliations:** 1 CSIRO Livestock Industries, Australian Animal Health Laboratory, Geelong 3220, Australia; 2 School of Life and Environmental Sciences, Deakin University, Geelong 3217, Australia

## Abstract

**Background:**

RNA polymerase III (pol III) type 3 promoters such as U6 or 7SK are commonly used to express short-hairpin RNA (shRNA) effectors for RNA interference (RNAi). To extend the use of RNAi for studies of development using the chicken as a model system, we have developed a system for expressing shRNAs using the chicken 7SK (ch7SK) promoter.

**Results:**

We identified and characterised the ch7SK promoter sequence upstream of the full-length 7SK small nuclear RNA (snRNA) sequence in the chicken genome and used this to construct vectors to express shRNAs targeting enhanced green fluorescent protein (EGFP). We transfected chicken DF-1 cells with these constructs and found that anti-EGFP-shRNAs (shEGFP) expressed from the ch7SK promoter could induce efficient knockdown of EGFP expression. We further compared the efficiency of ch7SK-directed knockdown to that of chicken U6 (cU6) promoters and found that the efficiency of the ch7SK promoter was not greater than, but comparable to the efficiency of cU6 promoters.

**Conclusion:**

In this study we have demonstrated that the ch7SK promoter can express shRNAs capable of mediating efficient RNAi in a chicken cell line. However, our finding that RNAi driven by the ch7SK promoter is not more efficient than cU6 promoters contrasts previous comparisons of mammalian U6 and 7SK promoters. Since the ch7SK promoter is the first non-mammalian vertebrate 7SK promoter to be characterised, this finding may be helpful in understanding the divergence of pol III promoter activities between mammalian and non-mammalian vertebrates. This aside, our results clearly indicate that the ch7SK promoter is an efficient alternative to U6-based shRNA expression systems for inducing efficient RNAi activity in chicken cells.

## Background

RNAi is a sequence-specific gene silencing mechanism [1] initiated by 19–29 nucleotide (nt) duplexes known as small-interfering RNAs (siRNAs) [2]. siRNAs are processed from long double-stranded RNA (dsRNA) molecules by the ribonuclease III enzyme Dicer [3] and are unwound and loaded as single-stranded RNAs into the RNA induced silencing complex (RISC) [4]. RISC can then silence gene expression via cleavage of messenger RNA (mRNA) transcripts that are complementary to the incorporated siRNA sequence [4]. RNAi-mediated silencing can be adapted for specific gene targets in vertebrates via transfection of siRNA duplexes [2] or DNA vectors which express siRNAs as short-hairpin RNAs (shRNAs) [5]. shRNAs are transcribed from these vectors as 19–29 nt inverted repeat sequences, separated by a 4–10 nt loop sequence and fold spontaneously to form hairpin structures, which are cleaved by Dicer into active siRNAs [5,6].

Pol III type 3 promoters are most commonly used to express shRNAs [6], as these promoters normally transcribe endogenous small-nuclear RNAs (snRNAs) such as U6 and 7SK. Termination of transcription by Pol III also occurs at defined tracts of 4–5 thymidines (T_4–5_) [7], which can be inserted downstream of shRNA coding sequences to ensure direct termination. Unlike type 1 and 2 promoters, pol III type 3 promoters are located entirely upstream of transcription start sites (+1) and feature characteristic promoter elements including; a TATA box beginning at around bp -20 (relative to +1), a Proximal Sequence Element (PSE) centred around bp -50 and a Distal Sequence Element (DSE) beginning around bp -240 [7]. In the human U6 and 7SK (h7SK) promoters, the DSE is comprised of at least one Octamer (OCT) motif [8,9] and an *Sph*I Post-octamer Homology (SPH) domain [10,11]. The DSE of the human 7SK (h7SK) also, contains an additional CACCC box enhancer located between the OCT and SPH elements [12]. U6 promoters are the most common type of promoter used for in vector-based shRNA expression systems, however, more recent approaches have preferred the use of 7SK promoters [13,14].

Given the recent completion of the chicken genome project [15], the adaptation and use of shRNA expression systems for RNAi in the chicken will be important for ensuing functional genomics studies. However, to date, most shRNA expression systems used in chickens feature mammalian pol III promoters. Although several chicken U6 (cU6) promoters have now been characterised and shown to drive efficient shRNA-mediated RNAi activity in chickens [16,17], recent work has highlighted that 7SK promoters in human (h7SK) and bovine (b7SK) can stimulate more efficient shRNA expression and RNAi activity than corresponding U6 promoters [13,14]. Given that expression of the 7SK snRNA appears to be conserved across non-mammalian and mammalian vertebrates [18] we sought to investigate whether a chicken 7SK promoter (ch7SK) would also confer greater levels of shRNA-mediated RNAi activity than the recently-characterised cU6 promoters.

## Results

### Identifying the chicken 7SK promoter

**Figure 1:**
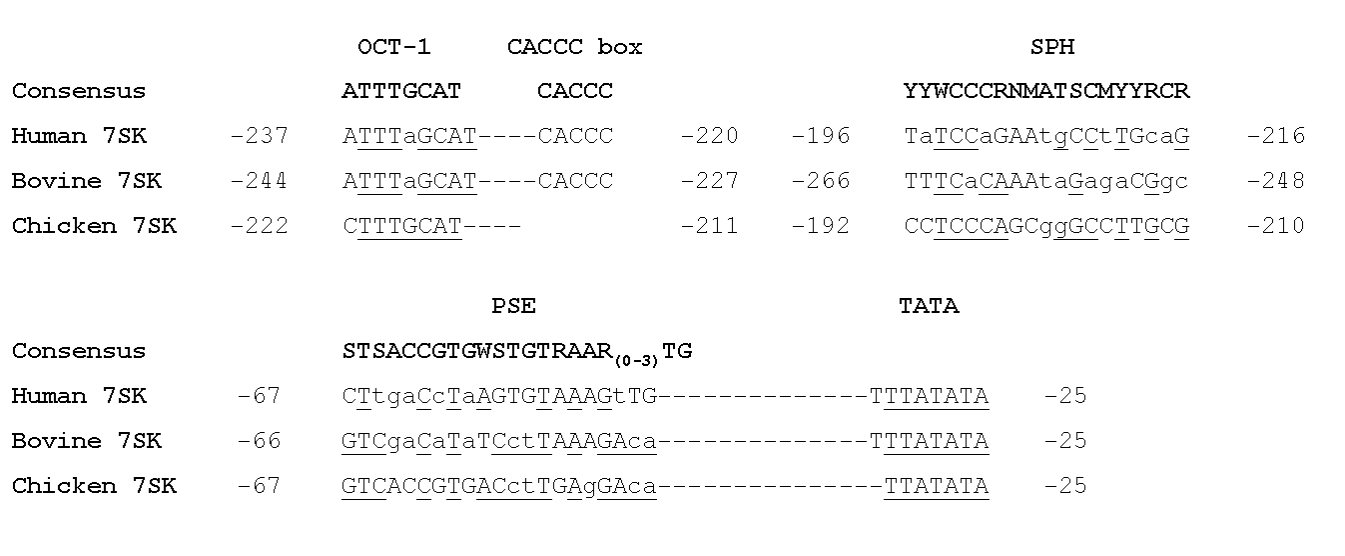
**Promoter element sequence alignment of chicken, bovine and human 7SK promoters**. The enhancer (DSE) of the chicken 7SK promoter contains OCT-1 and SPH motifs but no CACCC box. The basal promoter region features a PSE and TATA box with homology to consensus. Matches to the defined consensus sequences indicated at the top of the OCT-1 [21], SPH [12], PSE [20] and TATA sequences are shown in upper case. Nucleotides conserved between chicken and either bovine or human 7SK promoter elements are underlined. Nucleotide positions indicate the location (5' → 3') of each element in the promoter relative to the transcription start site (+1). Each dash mark between the OCT-1 and CACCC box and PSE and TATA box represents one nucleotide. Nucleotide abbreviations in consensus sequences are according to the International Union of Biochemistry convention for GENBANK.

Pol III type 3 promoters are characterised by the presence of gene-external promoter elements 5' of the transcription start site [7]. To identify the ch7SK promoter we used a bioinformatics approach and scanned the chicken genome for sequences with significant (80%) homology to the chicken 7SK snRNA gene sequence (GenBank Accession Number AJ890101). We then analysed the 5' flanking regions of these sequences for the presence of pol III promoter elements. The absence of sequences resembling a TATA box within the 5' flanking regions of 7SK snRNA BLAST hits on chromosomes 1, 2 and 4 (results not show), indicated that these 5' flanking regions probably did not encode functional 7SK pol III promoters. However, putative pol III promoter elements including a TATA box with homology to that of both the h7SK and b7SK promoters (Fig. 1), were present within the 5' flanking region of the full-length, ch7SK snRNA sequence, located on chicken chromosome 3 (Gga3, Contig NW_060336.1). This analysis indicated that the chicken genome contained only a single putative ch7SK promoter.

Using PCR we amplified a 783 bp region containing the putative ch7SK promoter sequence, which was cloned into pGEM^®^-T Easy. Sequencing of the cloned insert identified three clones; pch7SK-1 (783 bp), pch7SK-2 (782 bp) and pch7SK-3 (782 bp). Each of these clones showed 99% homology to the sequence immediately upstream of the ch7SK snRNA transcription start site on chromosome 3 of the chicken genome. Alignment of the last 300 bp (5' to 3') of each of these clone sequences against the h7SK [19] and b7SK [14] promoter sequences, identified typical pol III promoter elements including; a TATA box at bp -31 to -25, PSE at bp -67 to -46, and OCTmotifs at bp -222 to -215 (OCT-1), bp -138 to -132 (OCT-2a) and bp -97 to -90 (OCT-2b-not shown) and an SPH domain at bp -192 to -210 (Fig. 1). The PSE, OCT-1 and SPH elements also displayed considerable homology to published consensus sequences (Fig. 1) [20,21,11]. The presence of these elements within the cloned 5' flanking region of the ch7SK snRNA gene sequence, suggested that this region probably encoded a functional ch7SK promoter.

### The ch7SK promoter expresses shRNAs

**Figure 2:**
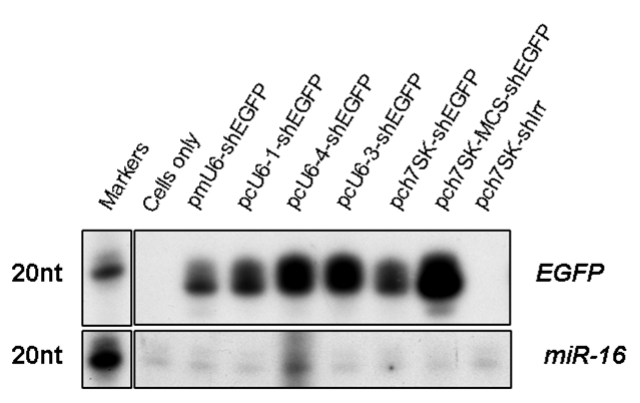
**Detection of shEGFP expression from ch7SK-shEGFP expression constructs in DF-1 cells**. DF-1 cells were transfected with shEGFP expression constructs as indicated above each lane. RNA samples were probed in solution with ^32^P-labelled shEGFP-specific LL91 RNA probe [14] and treated with RNAse A/T1. Protected shEGFP fragments were distinguished by comparison to RNA size markers (Decade™, Ambion).

In order to validate its function, the putative ch7SK promoter sequence was used to construct the shRNA expression vectors, pch7SK-shEGFP and pch7SK-MCS-shEGFP, designed to transcribe shRNAs targeting EGFP (shEGFP) (Supplementary Fig. 1). A third vector, pch7SK-shIrr, designed to transcribe an irrelevant shRNA (shIrr) targeting an influenza virus nucleocapsid protein (NP) [22] from the ch7SK promoter, was also constructed as a negative control (see Additional Files 1 and 2). The function of the isolated ch7SK promoter sequence was verified by detection of shEGFP expression in chicken embryo fibroblast (DF-1) cells transfected with the pch7SK-shEGFP or pch7SK-MCS-shEGFP constructs. RNA was extracted at 48 hours post-transfection and shEGFP expression was detected using an RNase protection assay (RPA) (Fig. 2). As a positive control for shEGFP detection, DF-1 cells were also transfected with vectors expressing identical shEGFP sequences from pre-validated mouse U6 (pmU6-shEGFP) [23], chicken U6-1 (pcU6-1-shEGFP), chicken U6-4 (pcU6-4-shEGFP) or chicken U6-3 (pcU6-3-shEGFP) pol III promoters [17].

A 19 nt band was detected in RNA samples from cells transfected with both the pch7SK-shEGFP and pch7SK-MCS-shEGFP constructs (Fig. 2). This band corresponded with the expected size of protected shEGFP sequence as well as specific bands detected in the positive control mouse U6 (mU6) and cU6-shEGFP-transfected positive control cells (Fig. 2). No shEGFP expression was detected in RNA samples from the pch7SK-shIrr negative control, or non-transfected cells (cells only) (Fig. 2). Detection of miR-16 as a loading control in all transfected and control RNA samples, confirmed the presence of total small RNAs (Fig. 2). These results verified the predicted promoter activity of the cloned ch7SK sequence and demonstrated that the ch7SK promoter could express shEGFP molecules.

### The ch7SK promoter directs shRNA-mediated RNAi knockdown

**Figure 3:**
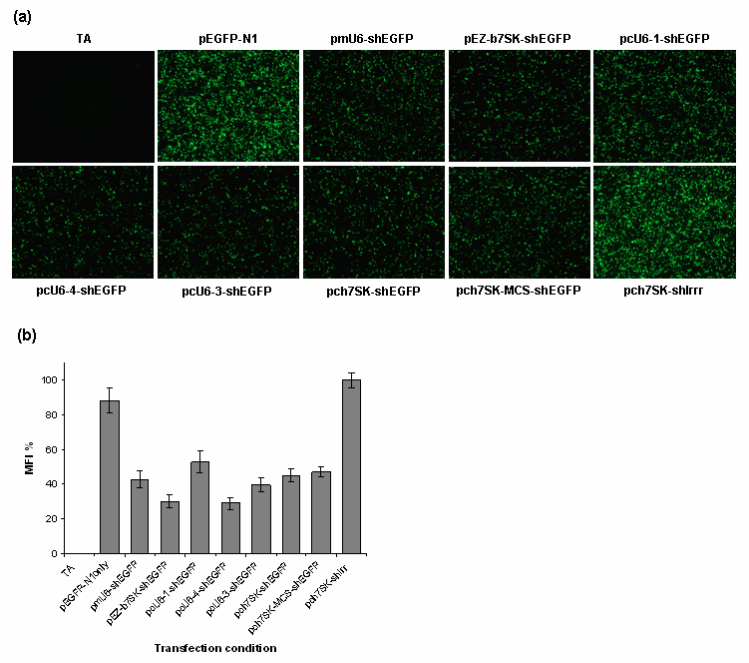
**EGFP knockdown conferred by chicken 7SK and U6 promoters in DF-1 cells**. (a) Fluorescence microscopy images of DF-1 cells transfected with pEGFP-N1 only, or co-transfected with pEGFP-N1 and various shEGFP expression plasmids as indicated for each image. TA is transfection reagent-only control (no-plasmid DNA). Images presented are representative of results from three independent experiments at 60 hours post-transfection (Magnification 50×). (b) Flow cytometry results for EGFP knockdown assays in co-transfected DF-1 cells. shEGFP expression constructs co-transfected with pEGFP-N1 are indicated on the *x *axis. EGFP knockdown was measured as a percent mean fluorescence intensity (MFI %), normalised to the average MFI of the negative control pch7SK-shIrr cells (100%). Error bars represent the standard error of the mean (SEM) calculated from at three independent experiments. Where no bars or error bars are visible the MFI and or SEM is less than 1%.

To verify that the shEGFP expressed by the ch7SK promoter could direct RNAi-mediated knockdown of an EGFP reporter gene, we conducted EGFP knockdown assays by co-transfecting chicken DF-1 cells with the pch7SK-shEGFP, pch7SK-MCS-shEGFP or positive control, pmU6-shEGFP and pEZ-b7SK-shEGFP [14] constructs, with an EGFP expression vector (pEGFP-N1) (Fig. 3). Given that co-transfection of reporter and shRNA expression plasmids is considered to be 100% efficient for validation of specific RNAi activity [24], we considered any reduction in EGFP fluorescence intensity to reflect RNAi-mediated EGFP knockdown. EGFP knockdown was assessed for each co-transfection condition in duplicate using fluorescence microscopy (Fig. 3a) and quantified using flow cytometry by sampling the mean fluorescence intensity (MFI) from triplicate co-transfections for each condition (See Materials & Methods) (Fig. 3b).

In DF-1 cells co-transfected with pEGFP-N1 and either the pch7SK-shEGFP or pch7SK-MCS-shEGFP constructs, the EGFP MFI was significantly reduced to 45.19% (± 3.37%) and 47.28% (± 3.14%) respectively (P < 0.001) (Fig. 3b and see Additional file 3). These reductions were not found to differ significantly from the EGFP MFI measured in the positive control pmU6-shEGFP (42.8% ± 4.67%) and pEZ-b7SK-shEGFP (45.27% ± 3.73%) co-transfected cells (P > 0.05) (Fig. 3b). Given both the mU6 and b7SK promoters are known to express functional shEGFP molecules that direct specific EGFP knockdown in DF-1 cells [17,23], this result indicated that the shEGFP molecules expressed by the ch7SK promoter could direct efficient knockdown (>50%) of EGFP in DF-1 cells.

### Comparison of ch7SK and cU6 promoter induced EGFP knockdown

We further compared the efficiency of RNAi knockdown mediated by the ch7SK promoter, to cU6 promoters; cU6-1, cU6-3 and cU6-4, [16,17] by comparing the reduction in EGFP MFI induced between the pcU6-1-shEGFP, pcU6-3-shEGFP and pcU6-4-shEGFP vectors and the two ch7SK-shEGFP constructs; pch7SK-shEGFP and pch7SK-MCS-shEGFP (Fig. 3).

Fluorescence microscopy results indicated that the EGFP knockdown induced by both of the ch7SK-shEGFP constructs was comparable to that induced by pcU6-4-shEGFP and pcU6-3-shEGFP, but greater than that conferred by pcU6-1-shEGFP (Fig. 3a). However, statistical analyses of MFI data (see Additional file 3) indicated no significant difference in the reduction of EGFP MFI between the pcU6-1-shEGFP (52.93% ± 6.25%), pch7SK-shEGFP (45.19% ± 3.37%), pch7SK-MCS-shEGFP (47.28% ± 3.15%) or pcU6-3-shEGFP (39.78% ± 3.93%) transfection conditions (P > 0.05) (Fig. 3b). The pcU6-4-shEGFP co-transfected cells showed the greatest reduction in EGFP MFI to 29.05% (± 3.26%), which was significantly lower than for the pch7SK-MCS-shEGFP and pcU6-1-shEGFP co-transfected cells (P = 0.05), but not significantly different to the MFI of either the pch7SK-shEGFP or pcU6-3-shEGFP-co-transfected cells (P > 0.05) (Fig. 3b). Taken together, these results indicated that neither of the ch7SK-shEGFP constructs induced more efficient RNAi-knockdown of EGFP than existing cU6-shEGFP constructs in DF-1 cells.

## Discussion

The chicken is an important livestock animal and a key model for studies of vertebrate development and gene function [25]. Thus the development of RNAi technologies adapted for use in chicken systems will be important for further annotation of the chicken genome. Although several recently characterised chicken U6 (cU6) promoters have been used to develop effective chicken-specific shRNA expression systems [16,17,26], 7SK promoters have been shown to direct more efficient RNAi activity than U6 promoters in mammals [13,14]. To investigate whether this promoter relationship also exists in chickens we have characterised the ch7SK promoter and compared its ability to confer RNAi activity to that of existing cU6 promoters.

Although several 7SK pseudogenes exist in the chicken genome [15] we characterised the ch7SK promoter sequence upstream of the full-length chicken 7SK snRNA sequence on chromosome 3. In the human, bovine and mouse genomes only single functional 7SK promoters have been identified upstream of 7SK snRNA coding sequences [19,14,27]. Our finding that only the full-length ch7SK snRNA sequence features an active upstream promoter region is therefore consistent with the notion that only a single functional 7SK promoter is present in the chicken genome. The ch7SK promoter was also found to contain typical pol III promoter elements; TATA, PSE, OCT and SPH (Fig. 1) which show positional and sequence similarities to those of the h7SK and b7SK promoters (Fig. 1) [19,14]. Further, we noted that the ch7SK locus was flanked by homologues of the glutathione S transferase-A3 and intestinal cell kinase (MAK-related kinase) genes which are also located 5' and 3' respectively of the identified mammalian 7SK loci. This level of synteny in the arrangement of the 7SK loci between chicken and other mammalian species is also reflected in recent comparative analyses of chromosome synteny blocks conserved between chicken and mammalian genomes [15]. This apparent conservation in the arrangement of the ch7SK locus with respect to surrounding genes indicates that the ch7SK promoter characterised in the present study may be the only functional 7SK promoter in the chicken genome.

Our results clearly demonstrated that the ch7SK promoter was able to express functional shRNA molecules capable of mediating greater than 50% RNAi-knockdown of the target EGFP reporter gene. However, we found no evidence that the ch7SK promoter could direct more efficient shRNA-mediated RNAi knockdown compared to the cU6-1, cU6-3 and cU6-4 promoters. This interpretation was based on a lack of significant difference in the level of EGFP MFI between cells co-transfected with pch7SK-shEGFP and any of the cU6-shEGFP constructs, or between cells co-transfected with pch7SK-MCS-shEGFP and pcU6-1-shEGFP and pcU6-3-shEGFP. We cannot rule out however, that the ch7SK promoter may actually be less efficient than the cU6-4 promoter, given that our MFI data indicated the pcU6-4-shEGFP construct could direct a more significant reduction in EGFP MFI than the pch7SK-MCS-shEGFP construct (Fig. 3b). Our results also reflect those of a recent study by Das et.al. [26], which compared the efficiency of cU6 and mU6 promoters for expression of a miRNA operon in DF-1 cells. The levels of luciferase knockdown induced by the cU6 promoters of chromosomes 18 and 28 (equivalent to cU6-1 and cU6-4 in our study respectively) are similar to those we have measured for these promoters against EGFP (Fig. 3b). Kudo and Sutou (2005) [16] and Wise et.al., (2007) [17] also report that the cU6-4 promoter shows the highest efficiency of the five cU6 promoters characterised so far; cU6-1, cU6-2, cU6-3, cU6-4 [16] and cU6-1variant [17]. Consideration of our results against these findings suggests therefore, that the ch7SK promoter may indeed be less efficient than the cU6-4 promoter. Given, however, that we could find no significant difference in the level of EGFP knockdown induced by shEGFP expressed from the ch7SK, cU6-1 and cU6-3 promoters, indicates that in general, the efficiency of the ch7SK promoter is comparable to that of these other cU6 promoters.

Interestingly, our results contrast findings published by Lambeth *et.al*., (2006) [14] and Koper-Emde *et. al*., (2004) [13], who independently demonstrated that the b7SK and h7SK promoters confer more efficient shRNA expression and RNAi activity than bovine and human U6 promoters, respectively. Despite close alignment of the ch7SK, h7SK and b7SK promoter elements, we noted some distinct differences within the DSE or enhancer region of the ch7SK promoter, which affect the structural organisation of the ch7SK promoter in relation to its mammalian homologues (Fig. 1). Given the structure and sequence of promoter elements within the DSE can influence maximal transcription efficiency in U6 and 7SK promoters [8,28,29], the variable structure of the ch7SK DSE may have an inherent impact upon its efficiency relative to U6 promoters.

Unlike the b7SK and h7SK promoters, the DSE of the ch7SK promoter does not contain a CACCC box (Fig. 1), which appears to be a distinct feature of 7SK promoters and is reported to serve an important role in enhancing the transcriptional activity of the h7SK promoter [12]. It is possible that the absence of a CACCC box in the ch7SK promoter may affect its overall efficiency by reducing enhancer activity in the ch7SK DSE to a level more similar to that seen in U6 promoters. This could explain why we observed comparable levels of EGFP knockdown induced between the ch7SK and cU6 promoters. Moreover, the absence of the CACCC box from the enhancer may further indicate that the enhancer mechanism in the ch7SK promoter may be more similar to that of U6 promoters than other mammalian 7SK promoters.

A second feature of the ch7SK enhancer, distinct from mammalian 7SK promoters is the presence of a C/A substitution at position 1 (bp -222) of the ch7SK OCT-1 motif (Fig. 1). Previous work has shown that mutation of the OCT-1 motif in the h7SK promoter has the strongest impact on transcriptional efficiency [29], so it is possible that this substitution may affect the activity of the ch7SK enhancer. However, an OCT-1 sequence identical to that of the ch7SK promoter is present in the enhancer of the RNA polymerase II (pol II) promoter of the chicken U4B (cU4B) snRNA [30] (see Additional files 4 and 5), which shows full affinity for the Octamer transcription factor (Oct-1) [31]. Therefore, it is unlikely that the ch7SK OCT-1 motif would affect promoter efficiency through a reduced ability to bind Oct-1. Optimal enhancer activity in the cU4B promoter however, is also dependent upon the presence of a downstream SPH domain adjacent to OCT-1 [31]. Interestingly, the position of the ch7SK SPH domain 4 bp downstream of OCT-1 corresponds closely to that of the cU4B promoter and shows striking homology (84%) to the cU4B SPH sequence (see Additional files 4 and 5) [30]. Given this level of structural identity, it is pertinent to suggest that the enhancer mechanism operating in the ch7SK promoter may be analogous to that of the cU4B promoter and require adjacent OCT-1 and SPH domains.

Co-dependence of OCT and SPH motifs in pol III enhancer mechanisms is common to other non-mammalian vertebrate pol III promoters including the Xenopus laevis tRNA^sec ^promoter [32]. Similarly, human U6 promoters also appear to rely upon the presence of both OCT and SPH elements for efficient enhancer activity [10]. This type of enhancer mechanism contrasts what is known about the function of the h7SK enhancer, where optimal transcription efficiency is not dependent upon the presence of an SPH domain [29]. Therefore, based on the structural features of the ch7SK enhancer, we propose that ch7SK enhancer mechanism may be less divergent from pol II and pol III promoters such as U4B and U6, than from other mammalian 7SK promoters.

Since ours is the first study to characterise the a non-mammalian vertebrate 7SK promoter, further characterisation and cross-species analysis of other vertebrate 7SK promoters would be necessary to evaluate this notion of divergent 7SK enhancer mechanisms. Whilst this type of investigation was beyond the scope of our study, it would go further to more strongly define promoter characteristics which govern the efficiency of 7SK promoters. This knowledge would be of substantial benefit to the development of more efficient shRNA expression systems for cross-species RNAi applications.

## Conclusion

In this study we have identified and isolated a functional chicken homologue of the 7SK snRNA promoter and demonstrated its ability to confer efficient shRNA expression and RNAi-knockdown of a reporter gene in a chicken cell line. We further found that the efficiency of the ch7SK promoter was similar to that of existing cU6 promoters, which contrasts previous comparisons of mammalian U6 and 7SK promoters. The ch7SK promoter is the first non-mammalian vertebrate 7SK promoter to be characterised, so this finding may reflect inherent differences in the divergence of pol III promoter activities between mammalian and non-mammalian vertebrates. This aside, our results clearly indicate that the ch7SK promoter is an efficient alternative to U6-based shRNA expression systems for inducing efficient RNAi activity in chicken cells. This and the characterisation of other chicken-specific promoters for RNAi applications will be of particular benefit to furthering functional genomic analysis of the chicken genome and in developmental studies which utilise the chicken as a model system.

## Methods

### Isolation of the ch7SK promoter from chicken genomic DNA

The ch7SK promoter sequence was amplified from chicken genomic DNA extracted from chicken embryo fibroblast (DF-1) cells (ATCC, CRL-12203) (Wizard^® ^Genomic DNA purification kit, Promega), using the primers: forward (TD245): 5'-GTCCAGCCATCCACCTCCCACCAATACTTC-3' and reverse (TD237): 5'-AAAGCTACGAGCTGCCCCAA-3'. Gradient PCR was conducted using; 9.5 ng of genomic DNA, 100 ng of each primer (TD245 & TD237), 2 mM MgCl_2 _(Qiagen), 250 μM dNTPs (Promega), 1 × PCR buffer (Qiagen) and 1 unit of *Thermus acquaticus *(*Taq*) polymerase (Promega), in a Mastercycler EP Gradient S thermocycler (Eppendorf AG). Cycle conditions were: 94°C – 5 minutes, 35 cycles of; 94°C – 1 minute, 69.4°C – 45 sec and 72°C – 1 minute, with a 5 minute final extension at 72°C.

A single PCR product of approximately 780 bp was amplified, purified using the Wizard SV PCR and Gel cleanup kit (Promega) and cloned using the pGEM^®^-T Easy vector cloning system (Promega). Ligations were transformed into TOP10F' *Escherichia coli *(*E. coli*) cells (Invitrogen) and plasmid DNA isolated from bacterial clones (QIAprep^® ^Spin Miniprep Kit, Qiagen) was sequenced (Micromon DNA sequencing facility, Monash University). Sequences were compared to public sequence databases using the mega-Basic Local Alignment Search Tool (mega-BLAST) [33]. The sequences of three clones containing the putative ch7SK promoter sequence; pch7SK-1 (783 bp), pch7SK-2 (782 bp) and pch7SK-3 (782 bp), were deposited into Genbank under the accession numbers, EF488955, EF488956 and EF488957 respectively.

### Construction of ch7SK-shRNA expression vectors

The pch7SK-shEGFP and pch7SK-shIrr expression vectors were constructed using the one-step PCR approach as described previously [17] (see Additional files 1 and 2). The primers used were; forward primer TD269 (5'-GAGGCTCAGTGTCACGCAGA-3') and reverse primer TD267 (5'-CTCGAGTTCCAAAAAAGCTGACCCTGAAGTTCATCTCTCTTGAAGATGAACTTCAGGGTCAGCAAAGCTACGAGCTGCCCCAA-3') (shEGFP) and TD268 (5'-CTCGAGTTCCAAAAAAGGATCTTATTTCTTCGGAGTCTCTTGAACTCCGAAGAAATAAGATCCAAAGCTACGAGCTGCCCCAA-3') (shIrr). pch7SK-shEGFP was amplified using pch7SK-3 template and pch7SK-shIrr was amplified using pch7SK-1 template.

The pch7SK-MCS-shEGFP vector was constructed from the pch7SK-MCS base-vector by ligation of complimentary annealed oligonucleotides (oligos) LL29 and LL30 as described previously [14] (see Additional files 1 and 2). The pch7SK-MCS base vector was constructed from pUC57 by ligating a 315 bp synthetic copy of the ch7SK promoter sequence between the *Eco*RI and *Hin*dIII sites (Celtek Genes). The ch7SK promoter sequence was altered between bp -5 to +11 to include a 3' multi-cloning site (MCS) comprising overlapping *Kpn*I, *Xho*I and *Eco*RI sites (see Additional files 1 and 2). All ligations were incubated at 4°C for 48 hours and transformed into TOP10F' *E. coli*.

### Sequence management and bioinformatics

Chicken genome sequence information was accessed through the National Centre for Biotechnology Information (NCBI) database [34] and viewed using the NCBI Map Viewer, *Gallus gallus *(chicken). Sequence alignments were performed using ClustalW [35] and Clone Manager 7 software (SciEd Central).

### Cell culture and transfection

Chicken DF-1 cells were maintained in 5% CO_2 _at 37°C in growth media as described previously [17] and harvested using 0.25% (w/v) trypsin-ethylenediaminetetraacetic acid (EDTA). Transfection of plasmid DNA for EGFP silencing assays was conducted in DF-1 cells grown to 80–90% confluence, in 8-well chamber slides (Nunc) for fluorescence microscopy or 24 well culture plates (Nunc) for flow cytometry. Cells were transfected with 500 ng or 1 μg of each plasmid, per well, for chamber slides or 24-well plates, respectively, using Lipofectamine™2000 transfection reagent (Invitrogen). For RNA extractions, DF-1 cells were grown in 25 cm^2 ^culture flasks (Corning) and transfected using 12.5 μg of plasmid and 25 μl of Lipofectamine™2000.

### Detection of shEGFP expression by RNase protection assay

RNA samples enriched for small RNAs (*mi*rVana miRNA isolation kit, Ambion) were purified from DF-1 cells 48 hours post-transfection of shEGFP expression plasmids. An RNAse protection assay (RPA) was conducted to detect expression of shEGFP (*mir*Vana Probe & Marker Kit, Ambion) using the RNA probe LL91 as described previously [14,17]. Expression of the miR-16 effector sequence was detected using the mir-16 RNA probe provided with the *mir*Vana Probe & Marker Kit, (Ambion). Duplicate RNA samples were prepared and probed separately for Probes were hybridised in-solution to duplicate RNA samples for detection of shEGFP and mir-16 expression. Mir-16 and shEGFP-probed RNA samples were RNase A/T1 treated according to the manufacturer's protocol (Ambion) and run on separate 15% polyacrylamide (8 M urea) gels. Gels were exposed to Medical X-ray film over 5 days at -80°C. Autoradiographs were developed using an FPM-100A X-ray processor (FUJIFILM).

### EGFP knockdown assays

EGFP expression was analysed at 60 hours post-transfection. Fluorescence microscopy was performed on duplicate co-transfections using a Leica DM LB Fluorescence Microscope (Leica Microsystems, Germany). Images were captured at 50× magnification using a Leica DC300F colour digital camera (Leica Microsystems, Germany) using Photoshop 7.0 imaging software (Adobe^®^). For flow cytometry, the EGFP fluorescence intensity was quantified as a mean fluorescence intensity (MFI) value for each co-transfection condition sampled in triplicate. Cells were harvested using 0.25% trypsin-EDTA, pelleted at 2000 rpm for 5 minutes, washed sequentially in cold phosphate buffered saline-A (PBSA) (Oxoid) and FACS-solution (PBSA + 1% FCS) and re-suspended in FACS-solution for sampling. Sampling and data acquisition was conducted using a FACScalibur (Becton Dickinson) fluorescence activated cell sorter and CELLQuest software (Becton Dickinson). The reduction in EGFP MFI for each co-transfection was calculated by normalising the average MFI from triplicate sampling, as a percentage of the MFI of the negative control shIrr/pEGFP-N1 co-transfected cells (100% ± 4.53% (SEM)) (Fig. 3b).

### Statistics

Normalised MFI data from three independent co-transfection experiments was analysed statistically by One-way Analysis Of Variance (ANOVA) and Tukey's multiple comparisons tests (Prism, GraphPad Software) (see Additional file 3). Significant difference in EGFP knockdown was accepted where P < 0.05.

## Authors' contributions

SB conducted PCR and cloning of ch7SK promoter, ch7SK expression vector construction, EGFP silencing assays and drafting of manuscript. TW constructed cU6 and mU6 expression vectors and participated in conducting RNase Protection Assays. DC contributed to experimental design. TD was involved in project conception, experimental design and drafting of the manuscript. All authors have read and approved the final manuscript.

## Supplementary Material

Additional file 1 Supplementary Figure 1. Shows construction of pch7SK-shEGFP and pch7SK-MCS-shEGFP expression vectors using one-step PCR and annealed oligo cloning respectively. Click here for file

Additional file 2 Figure legend for Supplementary Figure 1. Click here for file

Additional file 3 Supplementary Table 1. Shows P values for Tukey's statistical tests comparing EGFP MFI reductions for co-transfection conditions indicated in Figure 4b. Click here for file

Additional file 4 Supplementary Figure 2. Shows alignment of ch7SK and cU4B promoter enhancer regions. Click here for file

Additional file 5 Figure legend for Supplementary Figure 2. Click here for file

## References

[B1] Fire A, Xu S, Montgomery MK, Kostas SA, Driver SE, Mello CC (1998). Potent and specific genetic interference by double-stranded RNA in *Caenorhabditis elegans*. Nature.

[B2] Elbashir SM, Lendeckel W, Tuschl T (2001). RNA interference is mediated by 21- and 22-nucleotide RNAs. Genes Dev.

[B3] Bernstein E, Caudy AA, Hammond SM, Hannon GJ (2001). Role for a bidentate ribonuclease in the initiation step of RNA interference. Nature.

[B4] Hammond SM, Bernstein E, Beach D, Hannon GJ (2000). An RNA-directed nuclease mediates post-transcriptional gene silencing in Drosophila cells. Nature.

[B5] Brummelkamp TR, Bernards R, Agami R (2002). A system for stable expression of short interfering RNAs in mammalian cells. Science.

[B6] Amarzguioui M, Rossi JJ, Kim D (2005). Approaches for chemically synthesized siRNA and vector-mediated RNAi. FEBS Lett.

[B7] Schramm L, Hernandez N (2002). Recruitment of RNA polymerase III to its target promoters. Genes Dev.

[B8] Danzeiser DA, Urso O, Kunkel GR (1993). Functional characterization of elements in a human U6 small nuclear RNA gene distal control region. Mol Cell Biol.

[B9] Murphy S, Pierani A, Scheidereit C, Melli M, Roeder RG (1989). Purified octamer binding transcription factors stimulate RNA polymerase III – mediated transcription of the 7SK RNA gene. Cell.

[B10] Kunkel GR, Cheung TC, Miyake JH, Urso O, McNamara-Schroeder KJ, Stumph WE (1996). Identification of a SPH element in the distal region of a human U6 small nuclear RNA gene promoter and characterization of the SPH binding factor in HeLa cell extracts. Gene Expr.

[B11] Schaub M, Myslinski E, Schuster C, Krol A, Carbon P (1997). Staf, a promiscuous activator for enhanced transcription by RNA polymerases II and III. EMBO J.

[B12] Kleinert H, Bredow S, Benecke BJ (1990). Expression of a human 7S K RNA gene *in vivo *requires a novel pol III upstream element. EMBO J.

[B13] Koper-Emde D, Herrmann L, Sandrock B, Benecke BJ (2004). RNA interference by small hairpin RNAs synthesised under control of the human 7S K RNA promoter. Biol Chem.

[B14] Lambeth LS, Wise TG, Moore RJ, Muralitharan MS, Doran TJ (2006). Comparison of bovine RNA polymerase III promoters for short hairpin RNA expression. Anim Genet.

[B15] Hillier LW, Miller W, Birney E, Warren W, Hardison RC, Ponting CP, Bork P, Burt DW, Groenen MA, Delany ME, Dodgson JB, Chinwalla AT, Cliften PF, Clifton SW, Delehaunty KD, Fronick C, Fulton RS, Graves TA, Kremitzki C, Layman D, Magrini V, McPherson JD, Miner TL, Minx P, Nash WE, Nhan MN, Nelson JO, Oddy LG, Pohl CS, Randall-Maher J, Smith SM, Wallis JW, Yang SP, Romanov MN, Rondelli CM, Paton B, Smith J, Morrice D, Daniels L, Tempest HG, Robertson L, Masabanda JS, Griffin DK, Vignal A, Fillon V, Jacobbson L, Kerje S, Andersson L, Crooijmans RP, Aerts J, van der Poel JJ, Ellegren H, Caldwell RB, Hubbard SJ, Grafham DV, Kierzek AM, McLaren SR, Overton IM, Arakawa H, Beattie KJ, Bezzubov Y, Boardman PE, Bonfield JK, Croning MD, Davies RM, Francis MD, Humphray SJ, Scott CE, Taylor RG, Tickle C, Brown WR, Rogers J, Buerstedde JM, Wilson SA, Stubbs L, Ovcharenko I, Gordon L, Lucas S, Miller MM, Inoko H, Shiina T, Kaufman J, Salomonsen J, Skjoedt K, Wong GK, Wang J, Liu B, Wang J, Yu J, Yang H, Nefedov M, Koriabine M, Dejong PJ, Goodstadt L, Webber C, Dickens NJ, Letunic I, Suyama M, Torrents D, von Mering C, Zdobnov EM, Makova K, Nekrutenko A, Elnitski L, Eswara P, King DC, Yang S, Tyekucheva S, Radakrishnan A, Harris RS, Chiaromonte F, Taylor J, He J, Rijnkels M, Griffiths-Jones S, Ureta-Vidal A, Hoffman MM, Severin J, Searle SM, Law AS, Speed D, Waddington D, Cheng Z, Tuzun E, Eichler E, Bao Z, Flicek P, Shteynberg DD, Brent MR, Bye JM, Huckle EJ, Chatterji S, Dewey C, Pachter L, Kouranov A, Mourelatos Z, Hatzigeorgiou AG, Paterson AH, Ivarie R, Brandstrom M, Axelsson E, Backstrom N, Berlin S, Webster MT, Pourquie O, Reymond A, Ucla C, Antonarakis SE, Long M, Emerson JJ, Betran E, Dupanloup I, Kaessmann H, Hinrichs AS, Bejerano G, Furey TS, Harte RA, Raney B, Siepel A, Kent WJ, Haussler D, Eyras E, Castelo R, Abril JF, Castellano S, Camara F, Parra G, Guigo R, Bourque G, Tesler G, Pevzner PA, Smit A, Fulton LA, Mardis ER, Wilson RK (2004). Sequence and comparative analysis of the chicken genome provide unique perspectives on vertebrate evolution. Nature.

[B16] Kudo T, Sutou S (2005). Usage of putative chicken U6 promoters for vector-based RNA interference. J Reprod Dev.

[B17] Wise TG, Schafer DJ, Lambeth LS, Tyack SG, Bruce MP, Moore RJ, Doran TJ (2007). Characterisation and comparison of chicken U6 promoters for the expression of short hairpin RNAs. Anim Biotechnol.

[B18] Humphries P, Russell SE, McWilliam P, McQuaid S, Pearson C, Humphries MM (1987). Observations on the structure of two human 7SK pseudogenes and on homologous transcripts in vertebrate species. Biochem J.

[B19] Murphy S, Di Liegro C, Melli M (1987). The in vitro transcription of the 7SK RNA gene by RNA polymerase III is dependent only on the presence of an upstream promoter. Cell.

[B20] Dahlberg JE, Lund E The genes and transcription of the major small nuclear RNAs. Structure and Function of Major and Minor Small Nuclear Ribonucleoprotein Particles.

[B21] Sturm RA, Das G, Herr W (1988). The ubiquitous octamer-binding protein Oct-1 contains a POU domain with a homeo box subdomain. Genes Dev.

[B22] Ge Q, McManus MT, Nguyen T, Shen CH, Sharp PA, Eisen HN, Chen J (2003). RNA interference of influenza virus production by directly targeting mRNA for degradation and indirectly inhibiting all viral RNA transcription. Proc Natl Acad Sci USA.

[B23] Lambeth LS, Moore RJ, Muralitharan M, Dalrymple BP, McWilliam S, Doran TJ (2005). Characterisation and application of a bovine U6 promoter for expression of short hairpin RNAs. BMC Biotechnol.

[B24] Cullen BR (2006). Induction of stable RNA interference in mammalian cells. Gene Ther.

[B25] Stern CD (2005). The chick; a great model system becomes even greater. Dev Cell.

[B26] Das RM, Van Hateren NJ, Howell GR, Farrell ER, Bangs FK, Porteous VC, Manning EM, McGrew MJ, Ohyama K, Sacco MA, Halley PA, Sang HM, Storey KG, Placzek M, Tickle C, Nair VK, Wilson SA (2006). A robust system for RNA interference in the chicken using a modified microRNA operon. Dev Biol.

[B27] Moon IS, Krause MO (1991). Common RNA polymerase I, II, and III upstream elements in mouse 7SK gene locus revealed by the inverse polymerase chain reaction. DNA Cell Biol.

[B28] Boyd DC, Turner PC, Watkins NJ, Gerster T, Murphy S (1995). Functional redundancy of promoter elements ensures efficient transcription of the human 7SK gene *in vivo*. J Mol Biol.

[B29] Boyd DC, Greger IH, Murphy S (2000). In vivo footprinting studies suggest a role for chromatin in transcription of the human 7SK gene. Gene.

[B30] McNamara KJ, Walker RJ, Roebuck KA, Stumph WE (1987). Transcriptional signals of a U4 small nuclear RNA gene. Nucleic Acids Res.

[B31] Miyake JH, Botros IW, Stumph WE (1992). Differential protein-DNA interactions at the promoter and enhancer regions of developmentally regulated U4 snRNA genes. Gene Expr.

[B32] Myslinski E, Krol A, Carbon P (1992). Optimal tRNA((Ser)Sec) gene activity requires an upstream SPH motif. Nucleic Acids Res.

[B33] Altschul SF, Gish W, Miller W, Myers EW, Lipman DJ (1990). Basic local alignment search tool. J Mol Biol.

[B34] National Centre for Biotechnology Information. http://www.ncbi.nlm.nih.gov.

[B35] Chenna R, Sugawara H, Koike T, Lopez R, Gibson TJ, Higgins DG, Thompson JD (2003). Multiple sequence alignment with the Clustal series of programs. Nucleic Acids Res.

