# Supplementary figures and images for: Comparison of chicken 7SK and U6 RNA polymerase III promoters for short hairpin RNA expression
Source: BMC Biotechnol. 2007 Nov 19;7:79. doi: 10.1186/1472-6750-7-79 (PMC2235858; doi:10.1186/1472-6750-7-79)

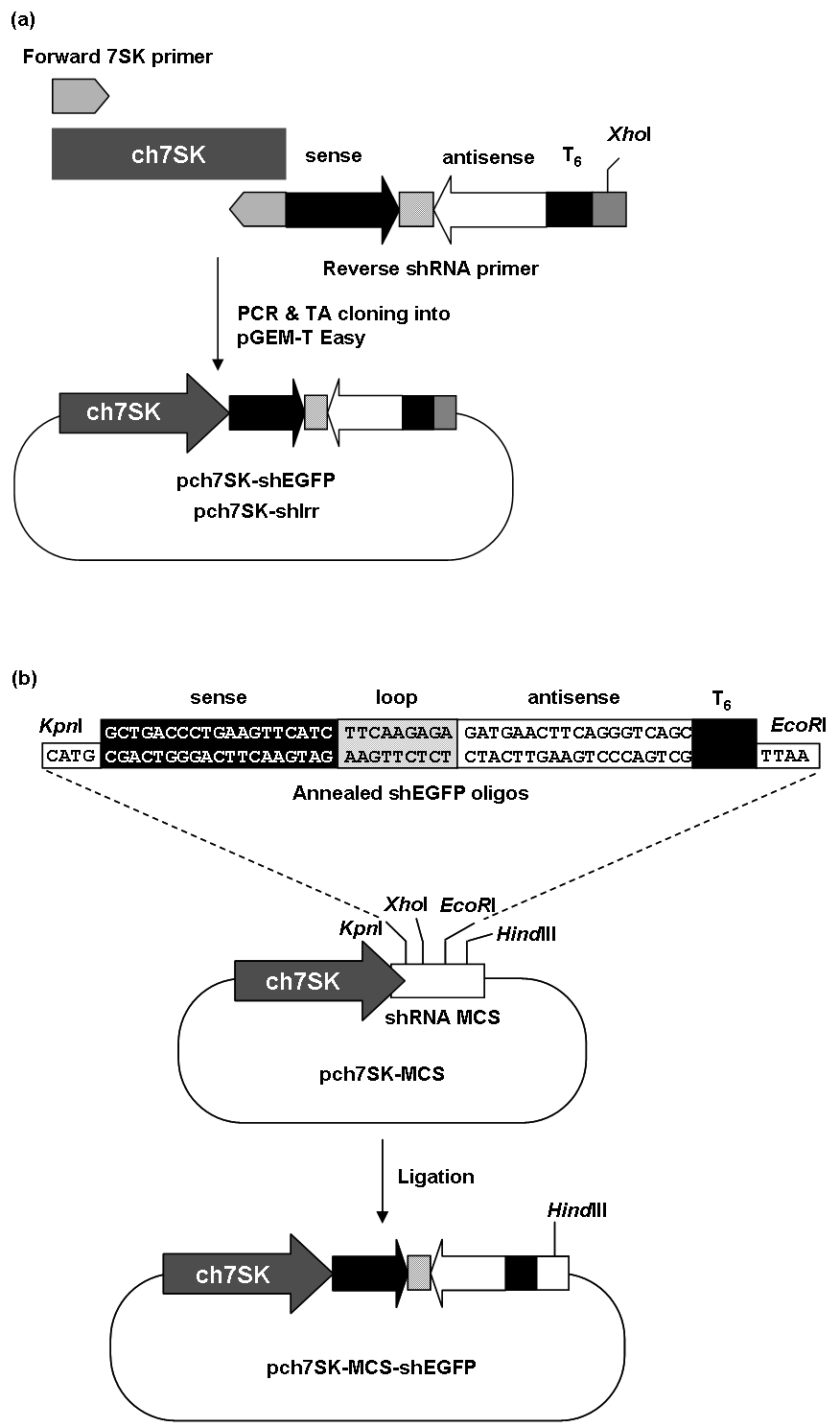

Supplement: Additional file 1 — Supplementary Figure 1. Shows construction of pch7SK-shEGFP and pch7SK-MCS-shEGFP expression vectors using one-step PCR and annealed oligo cloning respectively. [file 1472-6750-7-79-S1.tiff]

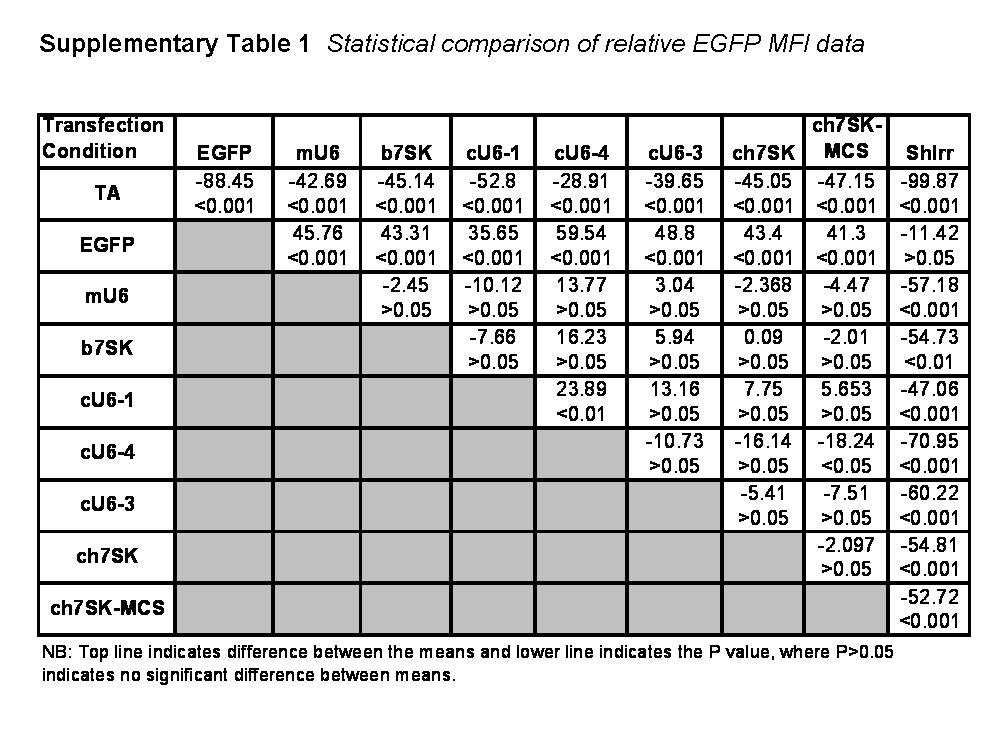

Supplement: Additional file 3 — Supplementary Table 1. Shows P values for Tukey's statistical tests comparing EGFP MFI reductions for co-transfection conditions indicated in Figure 4b. [file 1472-6750-7-79-S3.tiff]

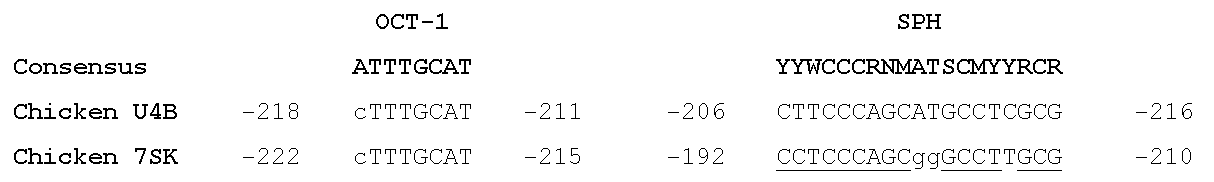

Supplement: Additional file 4 — Supplementary Figure 2. Shows alignment of ch7SK and cU4B promoter enhancer regions. [file 1472-6750-7-79-S4.tiff]
