# Supplementary material for: Comparison of chicken 7SK and U6 RNA polymerase III promoters for short hairpin RNA expression
Source: BMC Biotechnol. 2007 Nov 19;7:79. doi: 10.1186/1472-6750-7-79 (PMC2235858; doi:10.1186/1472-6750-7-79)
Supplement: Additional file 2 — Figure legend for Supplementary Figure 1. [file 1472-6750-7-79-S2.doc]

### **Supplementary Figure 1 - Construction of ch7SK-shEGFP** expression vectors.

(a) pch7SK-shEGFP and pch7SK-shIrr vectors were engineered using one-step PCR. Expression cassettes were amplified from cloned ch7SK promoter template using a forward primer (right-pointing grey arrow), to the cloned ch7SK promoter sequence (blue) and reverse primers (left-pointing grey arrow) overlapping the last 20bp of the promoter. The reverse primers also encoded the sense (black), loop [5] (light grey) and antisense (white) shRNA sequences, pol III terminator (Black, T_6_) and *Xho*I recognition sequence. (b) Construction of the pch7SK-MCS-shEGFP vector used an annealed oligonucleotide (oligo) cloning approach [148]. Complementary DNA oligos featuring the sense (black), loop [5] (grey) antisense (white), pol III terminator (T_6_), and *Kpn*I and *Eco*RI overhangs were annealed and ligated *Kpn*I/*Eco*RI into the 3’ multi-cloning site (MCS) of the pch7SK-MCS vector which contained a 315bp synthesised copy of the ch7SK promoter sequence.
