# Supplementary material for: Comparison of chicken 7SK and U6 RNA polymerase III promoters for short hairpin RNA expression
Source: BMC Biotechnol. 2007 Nov 19;7:79. doi: 10.1186/1472-6750-7-79 (PMC2235858; doi:10.1186/1472-6750-7-79)
Supplement: Additional file 5 — Figure legend for Supplementary Figure 2. [file 1472-6750-7-79-S5.doc]

### **Supplementary Figure 2** *Alignment of the enhancer regions of the chicken 7SK and U4B promoters***.**

Nucleotide positions of the OCT-1 and SPH element for the ch7SK and cU4B [30] promoters are given relative to the transcription start site (+1). The underscore indicates a shift in nucleotides for sequence alignment. Conserved nucleotide sequences are defined in upper-case.
